# Supplementation with Alpha-Tocopherol and Ascorbic Acid to Nonalcoholic Fatty Liver Disease's Statin Therapy in Men

**DOI:** 10.1155/2018/4673061

**Published:** 2018-05-17

**Authors:** Nikola Hadzi-Petrushev, Katerina Dimovska, Nikola Jankulovski, Dine Mitrov, Mitko Mladenov

**Affiliations:** ^1^Department of Physiology, Faculty of Natural Sciences and Mathematics, “Ss. Cyril and Methodius” University, Skopje, Macedonia; ^2^Department of Abdominal Surgery, Faculty of Medicine, “Ss. Cyril and Methodius” University, Skopje, Macedonia; ^3^Department of Radiology and Physical Therapy and Department of Internal Diseases in Ruminants, Faculty of Veterinary Medicine, “Ss. Cyril and Methodius” University, Skopje, Macedonia

## Abstract

Oxidative stress and inflammation contribute to the pathogenesis and progression of nonalcoholic fatty liver disease (NAFLD), and the control of lipid status by statins may help to stop the progression of NAFLD. We hypothesized that the addition of antioxidant vitamins C and E to atorvastatin therapy is associated with improved serum enzyme antioxidant status. NAFLD-related serum parameters and the activity of antioxidant enzymes, before and after 3 months of treatment, were determined in patients receiving atorvastatin alone or atorvastatin plus antioxidants. Compared to healthy controls, the patients, before receiving therapy, had increased catalase and glutathione reductase, with no significant difference in glutathione peroxidase activity. After the treatment, the levels of all three antioxidant markers were reduced to the same degree in both groups of patients, indicating therapy-induced lower level of reactive oxygen species production and/or improved nonenzymatic antioxidant mechanisms. Both therapies led to the normalization of the serum lipid profile and aminotransferase levels in the patients, but the reduction in CRP, although significant, did not reduce levels to those of the controls. The obtained results favor the notion that therapy with atorvastatin alone is equally efficient during the early stages of NAFLD, regardless of the addition of antioxidant vitamins. This trial is registered with TCTR20180425001.

## 1. Introduction

Nonalcoholic fatty liver disease (NAFLD) refers to the condition of hepatic steatosis in the absence of excessive alcohol consumption. It is the most common chronic, usually asymptomatic, liver disease that may progress to nonalcoholic steatohepatitis (NASH), fibrosis, cirrhosis, and hepatocellular carcinoma. The process begins with the liver becoming steatotic due to the accumulation of fat in liver cells resulting from the increased influx of free fatty acids (FFA) and/or de novo lipogenesis caused by abnormalities in energy metabolism [1]. At this point, the interactions between oxidative stress, inflammatory cytokines, and lipid peroxidation are the main contributors to the development of organelle dysfunction and inflammation within the liver tissue and progression of NAFLD and NASH [2]. The hepatocellular damage is indicated by elevated serum aminotransferase levels, and the increase in the C-reactive protein (CRP) could be used as a marker of inflammation and steatosis [3].

Oxidative stress plays one of the key roles in the development and pathogenesis of NAFLD since the levels of lipid peroxides are increased in both hepatic steatosis and NASH [1]. To cope with the reactive oxygen species (ROS), the cells use antioxidant enzymes and nonenzymatic antioxidant mechanisms, but when an imbalance between pro-oxidants and antioxidants is attained, cellular damages can occur [4]. Moreover, lipid peroxidation and ROS can lead to depletion of antioxidant enzymes. In patients with NASH, the pro-oxidation environment is related to decreased superoxide dismutase and glutathione peroxidase (GPx) activity, making the liver even more susceptible to oxidative damage [5].

There are no proven treatment guidelines and no single approved therapy for the treatment of NAFLD [6]. It has been shown that the control of cholesterol and triglyceride levels may help to stop the progression of NAFLD [7]. Therapy with atorvastatin in NAFLD patients with hyperlipidaemia was found to be both effective and safe, significantly reducing the serum aminotransferases and lipids [7, 8]. Based on the reported data [9] that damage from oxidative stress contributes to the progression of NAFLD and that it can be interrupted by inducing antioxidant pathways and suppressing proinflammatory cytokines, studies [10, 11] have examined the use of vitamin E alone or in combination with vitamin C, as additional therapy for NAFLD. However, the addition of antioxidant therapy to other types of NAFLD treatment may not always be associated with a greater beneficial effect. For example, one study showed that the addition of vitamins C and E for 24 months did not increase the efficacy of lifestyle intervention (with diet and increased physical activity), that alone led to significant improvement in liver histology [12]. Generally, clinical trials with vitamin E (as alpha-tocopherol) and other antioxidants often yield conflicting results due to their heterogeneity and focus mostly on the changes in liver enzymes and histology without evaluation of the antioxidant potential of the treatment [13, 14].

Hence, we hypothesized that the correction of hyperlipidaemia and the positive effects of atorvastatin would also be reflected on the enzymatic antioxidant status in the serum of the patients, elucidating the need/benefit of addition of vitamins E and C to the therapy.

## 2. Materials and Methods

### 2.1. Subjects

The present study was conducted on 40 male patients (median age 43, range 39–51 years; nonsmokers) diagnosed with NAFLD and a group of 34 healthy male individuals (median age 42, range 38–49 years; nonsmokers) used as controls. Written informed consent was obtained from all research subjects before their entry in the study. All experimental procedures were conducted in accordance with the International Ethical Guidelines for Biomedical Research Involving Human Subjects and the Helsinki Declaration (1964). A careful medical history for intake of drugs and alcohol was obtained, and detailed clinical examination was performed. Patients were excluded if they had any other chronic liver disease, malignancy, or inflammatory disease. None of the subjects had used medications known to precipitate steatohepatitis, and there was no history of treatment with lipid-reducing agents or vitamin supplements in 5 months prior to study entry.

During the process of diagnosis and selection, all subjects underwent abdominal ultrasonography to determine the presence/absence of the fatty liver. In order to be diagnosed of a fatty liver, the patients were required to have hepatorenal contrast and liver brightness [15]. The body mass index (BMI) was calculated with obesity defined as BMI > 30 kg/m^2^.

### 2.2. Procedure

The 40 patients diagnosed with NAFLD were randomly assigned to one of the two treatment groups using online random allocation software (QuickCalcs, GraphPad Software, San Diego, CA, USA). The healthy subjects and the patients diagnosed with NAFLD were grouped as follows: (1) control group consisting of 34 healthy subjects (Controls), (2) 20 patients diagnosed with NAFLD and later received atorvastatin (ATV), (3) 20 patients diagnosed with NAFLD and later received atorvastatin plus vitamins E and C (ATV + vitEC). The patients in the ATV group received orally 20 mg atorvastatin (Atoris) daily [16]. The patients in the ATV + vitEC group received orally 20 mg atorvastatin plus 400 IU of vitamin E and 1000 mg of vitamin C daily [17, 18]. Compliance was evaluated by history and pill count. No placebo was given to subjects not taking vitamin supplements. Blood was taken before treatment and again after a 3-month period.

### 2.3. Routine Laboratory

Fasting blood samples were drawn for the determination of serum aspartate aminotransferase (AST) and alanine aminotransferase (ALT) levels (NADPH kinetic UV test (Thermo Scientific, USA)) and lipid profile such as serum triglycerides (TG), total cholesterol (TC), low-density lipoprotein cholesterol (LDL-C), and high-density lipoprotein cholesterol (HDL-C) (enzymatic colorimetric test for TC, LDL-C, and HDL-C and photometric colorimetric test for TG (Human Diagnostics, Germany)). Levels of CRP were measured using turbidimetric immunoassay (Spectrum Diagnostics, Egypt).

### 2.4. Antioxidants

Serum catalase activity was measured as previously described by Goth [19]. Briefly, 50 *μ*l of the serum sample was incubated in 250 *μ*l of the substrate (65 *µ*M/ml H_2_O_2_ in 60 mM phosphate buffer, pH 7.4) at 37°C for 60 s. The enzymatic reaction was terminated with the addition of 250 *μ*l of 32.4 mM ammonium molybdate. The absorption by the H_2_O_2_-molybdate complex was measured at 405 nm against blanks. One unit of activity was defined as the decomposition of 1 *μ*M H_2_O_2_/min. The catalase activity is expressed as U/l.

The activity of GPx was determined according to Lawrence and Burk [20]. The activity was assayed by following the oxidation of NADPH at 340 nm for 3 min, at 25°C, in the presence of GR and reduced glutathione (GSH). The reaction mixtures containing 50 mM potassium phosphate, pH 7.0, 1 mM sodium azide, 2 mM GSH, 0.2 mM NADPH, 1 U/ml GR, 1.5 mM cumene hydroperoxide, and serum samples were incubated at 25°C for 5 min. The reaction was initiated by the addition of cumene hydroperoxide. One unit of activity was defined as the oxidation of 1 *μ*M of NADPH per min. Results are expressed as U/ml.

The rate of oxidation of NADPH by GSSG at 30°C was used as a measure of GR activity [21]. The reaction system contained 1 mM GSSG, 0.1 mM NADPH, 0.5 mM EDTA, 100 mM potassium phosphate buffer, pH 7.5, and serum sample. The oxidation of 1 *μ*M of NADPH per min was defined as a unit of glutathione reductase activity. The GR activity was expressed as U/ml.

### 2.5. Statistics

Results are presented as median and interquartile range (IQR). Wilcoxon matched-pair tests were used to compare the same groups at different time points (at study entry and after 3 months). The Kruskal–Wallis test was used to compare the medians of the three different groups at the same time point, and Dunn's multiple comparisons test was performed in selected instances to evaluate further differences between group pairs. All analyses were performed with GraphPad Prism 4.0 (GraphPad Software, San Diego, CA, USA). *p* < 0.05 was considered statistically significant.

## 3. Results

The median and IQR value of BMI was 31.0 (24.4–32.2) for the NAFLD patients (60% were obese) and 28.5 (23.7–30.9) for the controls (50% were obese). BMI remained within the same category for every subject during the study. Liver fat examined by ultrasonography did not change significantly after the treatment. No side effects were reported in this study.

### 3.1. Routine Biochemistry

At the study entry, serum aminotransferase levels were significantly higher in both groups of NAFLD patients (ATV and ATV + vitEC groups) compared to controls. The treatments with atorvastatin alone (ATV group) and atorvastatin plus vitamins (ATV + vitEC group) both led to a significant decrease in AST and ALT levels. The presence of inflammation in NAFLD was confirmed by the significantly higher CRP concentration in ATV and ATV + vitEC groups compared to controls at entry. Both therapies were successful in reducing the CRP level when comparing the respective medians for the ATV and ATV + vitEC groups, before and after treatment. However, at the end of the study, the concentration of CRP in treated groups was still significantly higher compared to the control group (Table 1).

At the beginning of the study, all NAFLD patients were characterized by dyslipidaemia. Compared to controls, before treatment, ATV and ATV + vitEC groups showed significantly higher TG and TC concentrations, while having increased LDL-C and significantly lower HDL-C. The treatment with atorvastatin alone and the combination of the statin and vitamins were equally successful in restoring the TG and LDL-C to levels that were significantly lower compared to the respective untreated groups of NAFLD patients. Furthermore, TG and LDL-C in the ATV and ATV + vitEC groups, following the 3 months of treatment, were not significantly different than the median concentration of the control group. The treatment with atorvastatin alone was effective in reducing the level of TC, whereas the concentration of TC in the ATV + vitEC group was not significantly different before and after treatment. In a similar, but opposite manner, only the combined therapy with atorvastatin and vitamins led to a significant increase in the level of HDL-C.

### 3.2. Antioxidants

**Figure 1:**
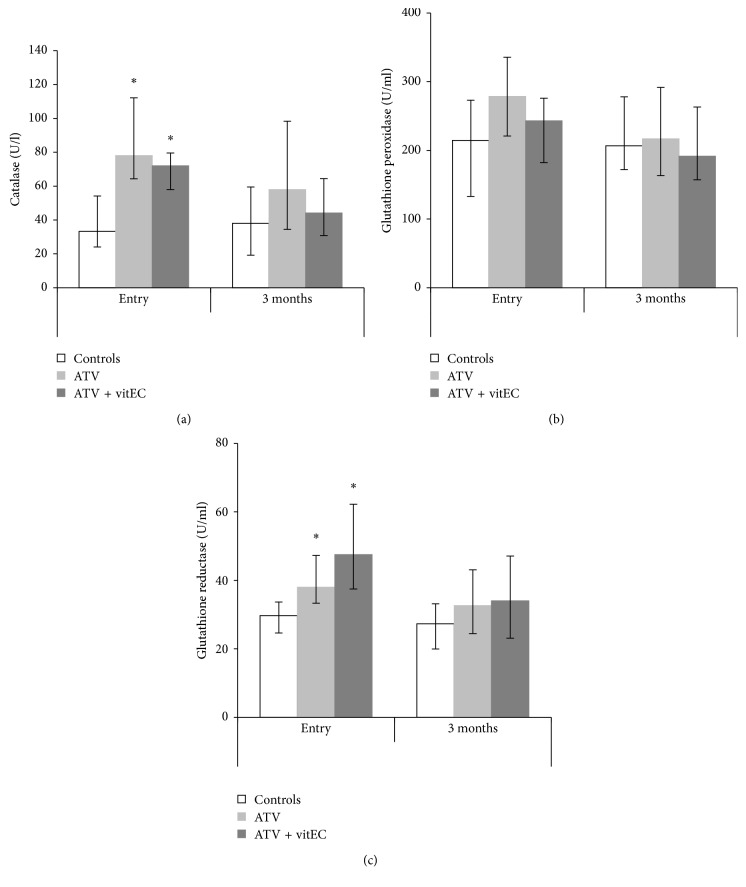
Activity (median and IQR) of antioxidant enzymes in the serum. ^∗^Comparing marked group versus control at the same time point; only cases where *p* < 0.05 are marked. (a) Catalase activity: controls versus ATV: *p*=0.003; controls versus ATV + vitEC: *p*=0.014. (b) Glutathione peroxidase activity. (c) Glutathione reductase activity: controls versus ATV: *p*=0.038; controls versus ATV + vitEC: *p*=0.008.

At the study entry, both groups of NAFLD patients had significantly higher catalase activity compared to the control group. Following the 3-month treatment period, these differences were reduced in relation to the control, but the catalase activity in ATV and ATV + vitEC groups was not significantly different compared to the respective groups at study entry. Regarding the enzymes that are part of the GSH redox cycle, our results showed no significant differences in the GPx activity in function of NAFLD at the study entry. Similarly, the two types of therapy did not cause significant change in GPx activity in any of the groups after the 3-month period. GR activity was significantly higher in the groups containing NAFLD patients compared to the control group at the study entry. After the treatment, both ATV and ATV + vitEC groups had reduced median GR activity that was not significantly different than the median of the appropriate control group. Still, the comparisons of medians before and after treatment in the case of the ATV and ATV + vitEC groups, respectively, revealed no significant differences regarding the GR activity in the serum (Figure 1).

## 4. Discussion

The observed dyslipidaemia in our NAFLD patients could be attributed to insulin resistance that leads to increased free fatty acid flux to the liver. The compensatory accelerated *β*-oxidation causes excessive electron flux in the electron transport chain and ROS overproduction [22]. When the cell's antioxidant capacity is exceeded, it leads to oxidative stress and ultimately apoptosis and release of cell contents [23]. We relate to the described processes in the increased AST and ALT levels observed in our NAFLD patients before treatment. The peroxidation products may also prompt immune responses and activate inflammatory pathways [24]. Although we did not measure proinflammatory cytokines, these might explain the elevated CRP levels in both groups of patients at study entry.

During the limited time span of the study (3 months), both therapies led to normalization of TG and LDL-C levels. TC was significantly reduced only in the ATV group, but the level of HDL-C was restored just in the patients receiving vitamins. The effects on the serum lipid profile could be associated with the ability of atorvastatin to occupy a portion of the binding site of HMG-CoA, leading to reduction of intrahepatic cholesterol that causes an increase in LDL receptor turnover [25]. Both therapies were also helpful in lowering the serum aminotransferase levels. This is in agreement with the studies of Athyros et al. [26] and Hyogo et al. [27], where statins were shown to improve liver enzymes in patients with NAFLD. In our ATV + vitEC group, the beneficial effect could also be related to the ability of vitamin E to prevent the development of NAFLD by ameliorating oxidative stress and hepatic apoptosis [28]. Regarding the observed decrease in serum CRP concentration, *in vitro* studies have shown that vitamin E inhibits the production of proinflammatory cytokines [29]. However, the improvement in liver enzymes and reduction of CRP levels after treatment in our ATV group of NAFLD patients could have been due to the anti-inflammatory and antioxidant effects of the statins that are independent of their lipid-lowering activity [30]. The inability of both applied therapies to reduce the CRP levels to values comparable to the healthy subjects might be explained in view of the unchanged BMI of the patients at the end of the study. Although the liver is known to be a major source of CRP, it is the accumulation of fat both in the adipose tissue and in liver steatosis that leads to increased CRP levels among obese patients [3].

As part of the adaptive response to the overproduction of pro-oxidants, cells increase the expression of genes involved in antioxidant defenses [31]. We speculate that this compensatory induction is responsible for the increased catalase and GR activity in the serum of our NAFLD patients before treatment. By catalyzing its removal, increased catalase also blunts the hydrogen peroxide's inhibitory effect on GR expression [32]. In spite of the increased GR activity observed at study entry in our NAFLD patients, the GPx activity in the serum was no different compared to controls, which may be related to stronger lipid peroxidation. Depletion in the hepatic GSH content may be the reason for this inappropriate GPx response, which is probably caused by the decreased synthesis of *S*-adenosylmethionine, the major methyl donor in the liver and precursor to GSH [33].

At the end of the study, the activities of antioxidant enzymes in control and treated groups were not significantly different compared to the respective groups at study entry. However, catalase and GR activity in treated groups were no longer increased compared to controls at the same time point. Hence, the tendency for reduction in the antioxidant enzyme activity in function of the therapy in our NAFLD patients could be explained by therapy-induced lower level of ROS production and/or improved nonenzymatic antioxidant mechanisms. The presence of oxidizable fat in the liver leads to peroxidation [34], and atorvastatin helps in reducing the fat contents consequently diminishing ROS production. The nonenzymatic antioxidant mechanisms could be complemented by the addition of the antioxidant vitamins E and C. Robertson et al. [31] reported that lower levels of nonenzymatic antioxidants are more likely the consequence than the cause on the oxidative stress observed in animal models of NASH, which in our case puts more emphasis on the decreased ROS production as an indirect effect of atorvastatin. However, during the limited time span of our experiment, the therapy-induced lower level of ROS production and/or improved nonenzymatic antioxidant mechanisms did not have significant effect on the enzymatic antioxidant potential in our NAFLD patients.

The evidence on the efficiency of vitamins is more ambiguous with some studies showing vitamins E and C to be a promising treatment of NAFLD [35], while others have shown them to be ineffective [12]. Kugelmas et al. [36] have shown that after an identical period of treatment as used in our study, there was no significant difference between the group of NASH patients that was submitted only to the diet and physical exercises and the group that received vitamin E. In the same manner, our results demonstrate that the therapy with atorvastatin alone as equally efficient for NAFLD, regardless of the addition of vitamins E and C to it. In some way, the data from our study put light on the dyslipidaemia, as the primary problem during the early stages of NAFLD, since addressing that situation with a lipid-lowering agent leads to beneficial effects even after a relatively short period of treatment. However, our results do indicate changes in the enzymatic antioxidant status in function of NAFLD, and obese subjects may be more susceptible to oxidative injury due to lower levels of circulating alpha-tocopherol [37]. Additionally, our data showed that a significant increase in the level of HDL-C was achieved only when using a combined therapy with atorvastatin and vitamins. Henceforth, we would certainly not recommend strict addition or exclusion of antioxidant therapy in the treatment of NAFLD with atorvastatin, a decision that should be outlined only upon consulting data from clinical studies and outweighing the risks [17] and benefits of vitamin supplementation.

## 5. Conclusions

In general, the results of this work show that the correction of hyperlipidaemia is also associated with the reduction of antioxidant enzyme activity in the serum of NAFLD patients. Hence, the study provides a potentially valuable insight to clinicians that the therapy with atorvastatin, in parallel to its effects on the dyslipidaemia, when supplemented by antioxidants has no different effects on the enzymatic antioxidant status in NAFLD patients.

## Figures and Tables

**Table 1:** Values (median and IQR) of measured serum parameters for each experimental group at study entry and after 3 months.

Examined parameter	Experimental groups		
	Controls	ATV	ATV + vitEC
*At study entry*			
AST (U/l)	23.7 (16.4–28.7)	42.7 (28.7–48.2) (*p*=0.009^*∗*^)	41.5 (32.5–53.5) (*p*=0.002^*∗*^)
ALT (U/l)	20.2 (14.5–28.4)	41.9 (29.8–69.7) (*p*=0.002^*∗*^)	49.7 (39.1–67.8) (*p* < 0.001^*∗*^)
CRP (mg/l)	0.8 (0.3–1.6)	6.2 (5.6–8.3) (*p* < 0.001^*∗*^)	6.9 (5.1–9.8) (*p* < 0.001^*∗*^)
TG (mmol/l)	1.2 (0.9–1.5)	3.2 (1.3–6.3) (*p*=0.018^*∗*^)	3.4 (1.6–5.5) (*p*=0.039^*∗*^)
TC (mmol/l)	4.6 (3.0–5.7)	7.6 (6.6–8.7) (*p*=0.006^*∗*^)	6.5 (5.9–7.3) (*p*=0.020^*∗*^)
LDL-C (mmol/l)	2.4 (1.6–3.2)	6.0 (4.5–6.9) (*p*=0.002^*∗*^)	5.1 (4.3–5.9) (*p*=0.004^*∗*^)
HDL-C (mmol/l)	1.5 (1.0–1.9)	0.7 (0.3–1.4) (*p*=0.047^*∗*^)	0.6 (0.3–0.9) (*p*=0.029^*∗*^)
*After 3 months*			
AST (U/l)	17.4 (15.0–24.1)	24.2 (20.1–25.3) (*p*=0.044^†^)	23.1 (18.7–33.7) (*p*=0.040^†^)
ALT (U/l)	23.8 (20.3–29.7)	30.8 (22.5–36.8) (*p*=0.004^†^)	31.2 (26.7–35.3) (*p*=0.002^†^)
CRP (mg/l)	1.1 (0.8–1.5)	3.2 (2.9–3.8) (*p*=0.003^*∗*^, *p*=0.031^†^)	3.1 (2.2–3.6) (*p*=0.011^*∗*^, *p*=0.004^†^)
TG (mmol/l)	1.8 (0.9–2.2)	1.3 (1.0–2.1) (*p*=0.002^†^)	1.2 (1.0–1.7) (*p*=0.007^†^)
TC (mmol/l)	4.8 (4.7–4.9)	4.8 (3.9–5.2) (*p*=0.009^†^)	5.7 (3.8–6.9)
LDL-C (mmol/l)	3.5 (2.2–3.9)	3.2 (2.9–3.4) (*p*=0.016^†^)	3.2 (3.1–3.3) (*p*=0.010^†^)
HDL-C (mmol/l)	1.2 (0.5–1.7)	1.5 (0.7–1.7)	1.6 (1.1–1.8) (*p*=0.015^†^)

^*∗*^Comparing marked group versus control at the same time point; ^†^comparing marked group versus same group at the study entry; only cases where *p* < 0.05 are marked.

## Data Availability

The data (excluding patient names) underlying the findings of the study could be obtained by contacting the corresponding author.
